# Pharmacokinetics, tolerability, and safety of TBI-223, a novel oxazolidinone, in healthy participants

**DOI:** 10.1128/aac.01542-24

**Published:** 2025-03-11

**Authors:** Antonio Lombardi, Fran Pappas, Paul Bruinenberg, Jerry Nedelman, Rajneesh Taneja, Dean Hickman, Maria Beumont, Eugene Sun

**Affiliations:** 1 Global Alliance for TB Drug Development 486654 , New York, New York, USA; 2 Vast Therapeutics 692781 , Morrisville, North Carolina, USA; St. George's, University of London, London, United Kingdom

**Keywords:** TBI-223, oxazolidinone, tuberculosis, pharmacokinetics, phase 1, antimicrobial safety

## Abstract

TBI-223 is an oxazolidinone antibiotic under clinical development for the treatment of tuberculosis. Preclinical data indicate potent antituberculosis activity and a potentially improved safety profile over linezolid. In a single-ascending dose study and a multiple-ascending dose study in 114 healthy adults, TBI-223 was generally safe and well tolerated at single doses up to 2,600 mg and multiple doses up to 2,400 mg daily over 14 days. No deaths, serious or severe adverse events, or discontinuations resulting from adverse effects (other than COVID-19) occurred. Except for two instances of orthostatic tachycardia in the single-ascending dose (SAD) study that resolved, no clinically significant electrocardiogram changes were noted. Concentration-QTc modeling found significant relationships of QTc with concentrations of TBI-223 and its main metabolite. However, groups receiving TBI-223 experienced QTc > 450 ms at a similar rate to those receiving placebo. TBI-223 exposures were nearly dose proportional. In the SAD study, TBI-223 exhibited a terminal half-life of 1.9–3.8 hours. Sustained-release tablets achieved a mean AUC_0-inf_ of 70%–80% relative to immediate-release tablets, with more than 50% lower mean *C*_max_. These results support further investigation of TBI-223 for the treatment of tuberculosis.

**CLINICAL TRIALS:**

This study is registered with ClinicalTrials.gov as NCT03758612 and NCT04865536.

## INTRODUCTION

In 2022, 1.3 million people died from tuberculosis (TB), and 7.5 million people were newly diagnosed. An estimated 410,000 people developed multidrug-resistant (MDR) or rifampin-resistant (RR) TB; of these, fewer than half were diagnosed and treated. The need for new and better therapies persists (1).

TBI-223 is a novel oxazolidinone in the same drug class as linezolid (Zyvox). Linezolid is approved in many countries around the world for drug-resistant, gram-positive bacterial infections. Antimicrobial effects result from the inhibition of protein synthesis in the ribosomes of the infecting organism (2). Studies of the bactericidal and sterilizing activity of linezolid in a mouse model of *Mycobacterium tuberculosis* (*Mtb*) infection have demonstrated that linezolid alone causes marked reductions in lung colony-forming units from mice following 1–3 months of therapy (3). The use of linezolid as part of a regimen for the treatment of TB has been studied in several trials (4–10). It is part of the BPaL(M) regimen recommended by the World Health Organization (WHO) for rifampicin-resistant/MDR-TB and a WHO Group A drug for RR/MDR-TB (11, 12). However, adverse events (AEs), especially peripheral neuropathy and myelosuppression, challenge the treatment success of combination regimens with linezolid. Although these AEs are often manageable with dose reduction or pausing, a need remains to develop effective and tolerable oxazolidinones with a better safety profile than linezolid.

TBI-223 is a drug candidate resulting from TB Alliance’s efforts to develop a safer oxazolidinone with the potential to deliver efficacy similar to linezolid but without its bone marrow and neurologic toxicities. TBI-223 showed potent *in vitro* activity and efficacy in mouse models of TB as a single agent and in combination with bedaquiline and pretomanid (13). In a murine dose-fractionation study, both the area under the curve (AUC) of TBI-223 concentration vs time and time over a dosing interval where the concentration was above the minimum inhibitory concentration (MIC) (TMIC) were identified as efficacy drivers (internal data).

Mammalian mitochondrial protein synthesis (MPS) inhibition is the presumed reason for adverse events associated with long-term administration of linezolid, such as anemia, thrombocytopenia, and peripheral neuropathy (14, 15). TBI-223 was selected for preclinical development based on reduced MPS inhibition in HepG2 cells. It had a greater than sixfold higher half-maximal inhibitory concentration (IC_50_; >27 µg/mL) and greater selectivity index, as assessed by MPS IC_50_ over *Mtb* MIC_50_, compared with linezolid (16), suggesting a potentially lower risk of adverse events associated with MPS inhibition. Supporting this hypothesis, pivotal Good Laboratory Practice toxicity studies in rats and dogs of up to 3 months’ duration showed only mild and reversible changes in erythroid parameters at the highest dose, without corresponding changes in bone marrow histology or cytology (internal data). However, at higher dose levels and exposures with repeated administration in dogs, prolonged QTc intervals were observed. Also observed were reversible nervous system-related clinical signs—tremors, abnormal gait, and convulsions, which were distinct from the neurodegeneration associated with longer-term administration of linezolid.

We report here the first clinical trial of TBI-223, CL-001 (NCT03758612), a randomized, placebo-controlled, partially blinded, single-ascending dose (SAD) study with food-effect assessments. The objectives were to evaluate the safety, tolerability, and pharmacokinetics (PK) of TBI-223 oral suspension, TBI-223 oral enteric capsules, and TBI-223 tablet formulations.

We are also reporting the second clinical study of TBI-223, CL-002 (NCT04865536), a randomized, placebo-controlled, partially blinded, multiple-ascending dose (MAD) study. The objectives of CL-002 were to evaluate the safety, tolerability, and PK of single and multiple doses of TBI-223 administered as sustained-release (SR) formulation tablets or as a combination of SR and immediate-release (IR) formulation tablets in healthy adult participants under fed and fasted conditions.

## RESULTS

### Enrollment

#### CL-001

Reporting groups and numbers of participants enrolled per group are shown in Table 1. All participants dosed in each reporting group completed all study procedures for that group, although PK exposure metrics from two participants in group 7 were not included in summary statistics because of early emesis. One subject from group 5a withdrew from group 5b before dosing because of a positive drug screen.

**TABLE 1:** Reporting groups in SAD study CL-001, all single dose

Group	Details
0	Placebo oral suspension, fasting, *n* = 14. For each of the seven reporting groups, 1, 2, 3a, 4, 5a, 6, and 7, in addition to the *n* participants indicated below for those groups randomized to TBI-223, two participants were randomized to placebo. The two participants randomized to placebo along with reporting group 5a also received placebo along with reporting group 5b.
1	50 mg TBI-223 oral suspension, fasting, *n* = 6
2	100 mg TBI-223 oral suspension, fasting, *n* = 6
3a	300 mg TBI-223 oral suspension, fasting, *n* = 6
3b	300 mg TBI-223 enteric capsule, fasting, *n* = 6 as *n* = 4 from group 3a and *n* = 2 newly enrolled^*a*^
4	600 mg TBI-223 oral suspension, fasting, *n* = 6
5a	1,200 mg TBI-223 oral suspension, fasting, *n* = 8
5b	1,200 mg TBI-223 oral suspension, fed, *n* = 7 from group 5a^*b*^
6	2,000 mg TBI-223 oral suspension, fasting, *n* = 6
7	2,600 mg TBI-223 oral suspension, fasting, *n* = 8
8a	1,800 mg TBI-223 sustained-release tablet, variant SR1, fed, *n* = 6
8b	1,800 mg TBI-223 sustained-release tablet, variant SR2, fed, *n* = 6
8c	1,800 mg TBI-223 sustained-release tablet, variant SR3, fed, *n* = 6
9a	2,000 mg TBI-223 immediate-release tablet, fasted, *n* = 6
9b	2,000 mg TBI-223 immediate-release tablet, fed, *n* = 6 from group 9a

^*a*^ It was hoped to enroll the same participants from 3a into 3b. However, two participants opted not to continue. Two additional participants were enrolled to reach a total of six.

^*b*^ It was hoped to enroll the same participants from 5a into 5b. However, one subject was withdrawn for a positive urine drug screen. It was decided not to replace that subject.

#### CL-002

Reporting groups and numbers of participants enrolled per group are shown in Table 2. All participants in groups 0a, 0b, and 1 received all 15 intended doses. Five participants in group 2a received all 15 intended doses. Four participants in group 2a withdrew before completing the 14 fed doses because of COVID-19. Four replacements were enrolled into group 2b, and all four received all 14 intended fed doses (they did not receive the initial fasted dose).

**TABLE 2:** Reporting groups in MAD study CL-002^*a*^

Group	Details
0a	3× placebo SR1 600 mg tablets, *n* = 3 Single dose fasting Day 1, multiple doses fed Days 4–17 Randomized along with group 1
0b	3× placebo SR1 600 mg tablets + 1× Placebo IR 600 mg tablet, *n* = 3 Single dose fasting Day 1, multiple doses fed Days 4–17 Randomized along with group 2a
1	1,800 mg TBI-223 as 3× SR1 600 mg tablets, *n* = 9 Single dose fasting Day 1, multiple doses fed Days 4–17
2a	2,400 mg TBI-223 as 3× SR1 600 mg tablets + 1× IR 600 mg tablet, *n* = 9 Single dose fasting Day 1, multiple doses fed Days 4–17 Four of the nine participants did not complete the 14 planned multiple doses because of COVID This group was used for fed (Day 4) vs fasted (Day 1) comparison
2b	2,400 mg TBI-223 as 3× SR1 600 mg tablets + 1× IR 600 mg tablet, *n* = 5 from group 2a plus *n* = 4 newly enrolled, replacement participants The replacement participants received multiple doses fed Days 1–14 They did not receive a single dose under fasting conditions This group was used for comparison of exposure after 14 fed doses to exposure after one fed dose and for evaluation of steady state

^*a*^ In all cases, all tablets comprising the treatment were administered at the same time.

### Demographics

The demographic characteristics by study and dose group in CL-001 and CL-002 are shown in Table 3. Groups were generally balanced, with some differences because of small sample sizes.

**TABLE 3:** Demographics of participants in SAD study CL-001 and MAD study CL-002

Group			Age (years)		Weight (kg)		Female	Hispanic or Latino^*a*^	White^*b*^	Black or Afr. Amer.^*b*^
		*n*	Mean	Range	Mean	Range	%	%	%	%
CL-001		86	34.1	19, 49	76.44	55.7, 104.7	48.8	50.0	62.8	33.7
0	Placebo susp, fasted	14	35.1	21, 47	75.21	60.4, 94.1	14.3	57.1	57.1	35.7
1	50 mg susp, fasted	6	34.5	23, 47	79.85	57.3, 102.4	50.0	33.3	16.7	66.7
2	100 mg susp, fasted	6	32.3	28, 39	80.22	65.9, 96.8	33.3	50.0	66.7	33.3
3a	300 mg susp, fasted	6	27.2	19, 36	68.65	55.7, 90.0	83.3	66.7	66.7	33.3
3b	300 mg enteric, fasted^*c*^	6	31.2	19, 42	74.53	64.4, 90.0	66.7	50.0	50.0	50.0
4	600 mg susp, fasted	6	37.2	27, 49	71.07	56.7, 82.1	66.7	66.7	66.7	33.3
5a	1,200 mg susp, fasted	8	32.6	21, 44	83.31	62.4, 104.7	62.5	37.5	50.0	37.5
5b	1,200 mg susp, fed^*d*^	7	32.7	21, 44	82.94	62.4, 104.7	57.1	42.9	42.9	42.9
6	2,000 mg susp, fasted	6	38.7	24, 47	78.47	62.1, 93.7	33.3	16.7	50.0	50.0
7	2,600 mg susp, fasted	8	32.8	22, 45	77.80	61.5, 103.9	62.5	62.5	87.5	12.5
8a	1,800 mg SR1, fed	6	39.2	27, 49	77.48	63.8, 83.9	66.7	33.3	50.0	50.0
8b	1,800 mg SR2, fed	6	30.7	25, 38	78.47	62.3, 88.4	66.7	33.3	100.0	0
8c	1,800 mg SR3, fed	6	34.2	24, 46	72.67	58.1, 92.6	66.7	66.7	66.7	33.3
9a and 9b	2,000 mg IR tab, fasted	6	33.3	22, 43	73.85	58.6, 93.5	16.7	66.7	83.3	16.7
CL-002		28	37.4	22, 50	76.5	55.7, 92.9	50.0	32.1	35.7	60.7
0a and 0b	Placebo	6	41.0	29, 50	78.2	61.9, 89.2	50.0	16.7	50.0	33.3
1	1,800 mg	9	35.8	23, 47	75.0	56.8, 92.9	44.4	44.4	44.4	55.6
2a and 2b	2,400 mg	13	36.8	22, 50	76.8	55.7, 91.2	53.8	30.8	23.1	76.9

^*a*^ Hispanic or Latino is a category of ethnicity; its complement is Not Hispanic or Latino.

^*b*^ White and Black or African American (Afr. Amer.) are categories of race. Their complement comprises Asian, African Indian or Alaska Native, and Multiracial.

^*c*^ Four participants from 300 mg susp, fasted, plus two newly enrolled.

^*d*^ Seven participants from 1,200 mg susp, fasted.

### Pharmacokinetics

Concentrations of TBI-223 and its metabolite, M2, were measured in CL-001 and CL-002.

#### CL-001

**Fig 1:**
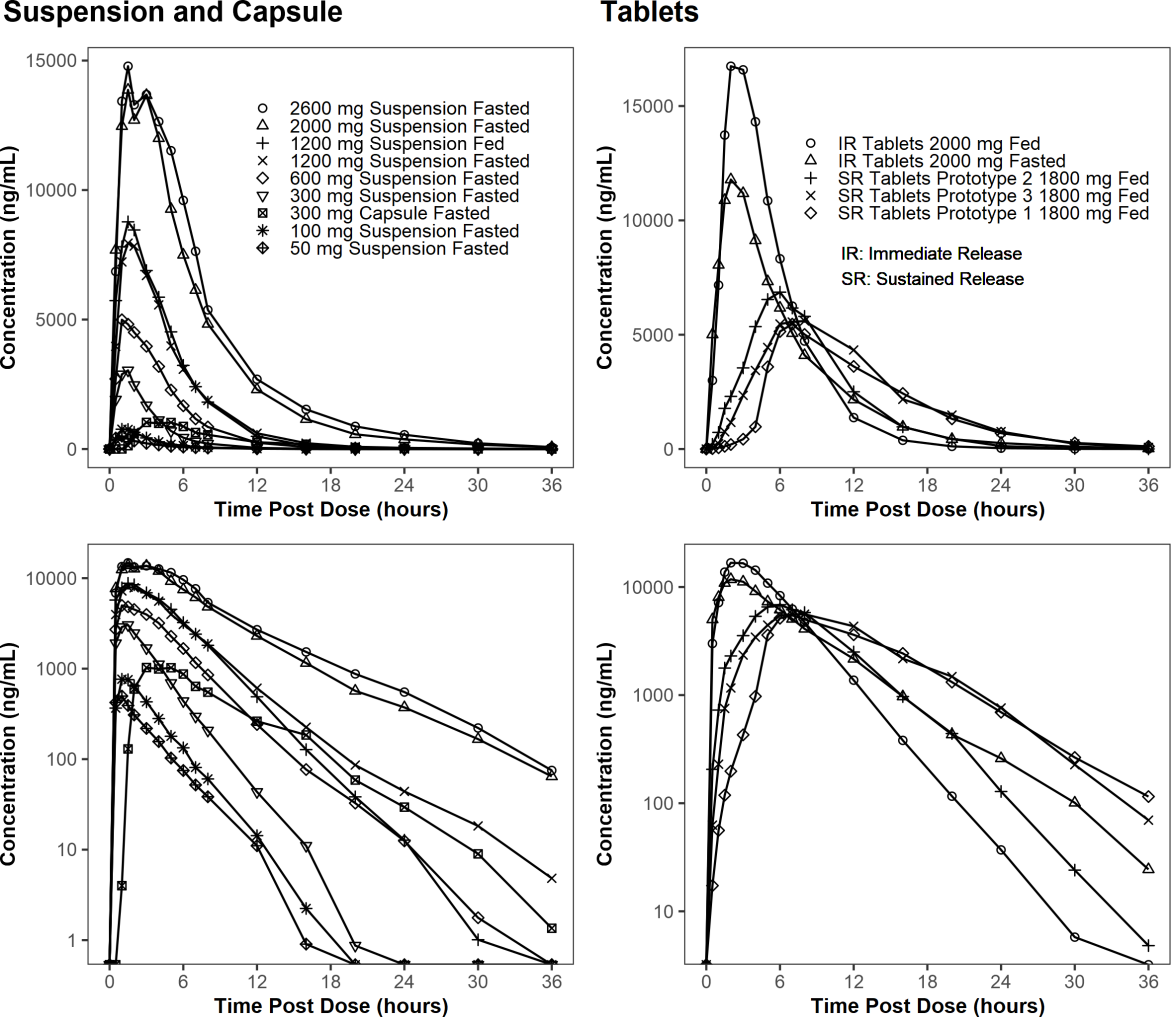
Mean plasma concentration-time profiles of TBI-223 after single doses of TBI-223 oral suspension, capsule, and tablet administered under fasted and fed conditions on linear and logarithmic scales.

The mean concentration profiles of TBI-223 are shown in Fig. 1. PK parameters are summarized in Table 4. For M2, see Fig. S1 and Table S1.

**TABLE 4:** TBI-223 exposure metrics in SAD study CL-001^*a*^

Group		*T*_max_ (h)	*C*_max_ (µg/mL)	AUC_0-*t*_ (μg·h/mL)	AUC_0-inf_ (μg·h/mL)	*T*_1/2_ (h)	CL/F (L/h)	*V*_*Z*_/F (L)
	*n*	Median (range)	Mean (SD)	Mean (SD)	Mean (SD)	Mean (SD)	Mean (SD)	Mean (SD)
Suspension and enteric capsule								
50 mg susp, fasted	6	1.00 (0.50, 1.00)	0.506 (0.156)	1.62 (0.343)	1.64 (0.344)	2.19 (0.239)	31.6 (7.09)	101 (29.0)
100 mg susp, fasted	6	1.50 (1.00, 2.00)	0.811 (0.201)	2.73 (0.631)	2.76 (0.628)	1.96 (0.165)	38.0 (8.94)	107 (23.2)
300 mg susp, fasted	6	1.37 (1.00, 1.53)	3.09 (0.664)	10.7 (2.09)	10.7 (2.10)	1.94 (0.238)	28.9 (5.28)	81.8 (23.8)
300 mg enteric, fasted	6	5.00 (3.00, 16.0)	1.36 (0.610)	8.64 (1.69)	8.69 (1.69)	3.43 (1.02)	35.8 (8.40)	179 (71.8)
600 mg susp, fasted	6	1.75 (1.00, 4.00)	5.80 (2.40)	25.5 (9.56)	25.5 (9.58)	2.63 (0.693)	25.7 (7.31)	96.9 (37.1)
1,200 mg susp, fasted	8	1.50 (1.00, 3.02)	8.53 (1.03)	45.9 (13.0)	45.0 (13.8)	2.93 (0.706)	28.5 (7.56)	119 (33.3)
1,200 mg susp, fed	7	1.50 (0.50, 3.00)	9.15 (2.74)	47.6 (14.3)	47.7 (14.3)	2.32 (0.158)	27.1 (7.82)	90.6 (26.8)
2,000 mg susp, fasted	6	1.52 (1.00, 3.05)	14.8 (2.65)	107 (17.8)	107 (17.8)	3.81 (0.870)	19.2 (3.14)	104 (19.2)
2,600 mg susp, fasted	6	2.25 (1.50, 5.00)	16.2 (4.64)	127 (37.7)	127 (37.8)	2.93 (0.534)	21.9 (5.80)	92.6 (31.3)
Tablets								
1,800 mg SR1, fed	6	7.00 (6.00, 16.0)	7.04 (2.01)	71.0 (18.9)	71.3 (18.8)	3.70 (0.271)	30.5 (11.3)	162 (120)
1,800 mg SR2, fed	6	6.50 (4.00, 8.00)	9.58 (3.39)	69.4 (16.0)	69.5 (16.0)	2.32 (0.581)	30.1 (6.67)	96.1 (5.37)
1,800 mg SR3, fed	6	7.50 (5.00, 20.0)	8.49 (1.67)	82.6 (19.4)	82.6 (19.4)	3.08 (0.784)	25.3 (5.76)	115 (41.7)
2,000 mg IR tab, fasted	6	2.00 (1.00, 3.00)	12.8 (2.32)	86.3 (21.2)	86.4 (21.2)	2.80 (0.604)	24.2 (5.42)	97.5 (27.1)
2,000 mg IR tab, fed	6	3.00 (1.00, 4.00)	19.7 (5.64)	100 (27.3)	100 (27.3)	2.38 (0.101)	21.0 (5.04)	71.9 (17.0)

^*a*^ *C*_max_ and AUCs for SR-1, SR-2, and SR-3 were normalized to the 2,000 mg dose; e.g., reported *C*_max_ = observed *C*_max_ × (2,000/1,800). Curves in Fig. 1 are not so normalized. Exposure metrics from two participants at 2,600 mg suspension, fasted, were excluded from summary statistics because of early emesis. susp: suspension. SR: Sustained Release. IR: Immediate Release. tab: tablet. *T*_max_: time of the maximum plasma concentration. *C*_max_: maximum concentration. AUC_0-*t*_: area under the plasma concentration-time curve from time zero to the time of the last quantifiable concentration, as calculated by the linear trapezoidal rule. AUC_0-inf_: area under the plasma concentration-time curve from the time of dosing extrapolated to infinity. *T*_1/2_: terminal elimination half-life. CL/F: apparent total plasma clearance after an oral dose. *V*_*z*_/F: apparent volume of distribution after an oral dose.

TBI-223 mean half-life (*T*_1/2_) was 1.94–3.81 hours across the 14 reporting groups. Median time to the maximal concentration (*T*_max_) was 1.00–2.25 hours for the oral suspension, 2.00–3.00 hours for the IR tablet, 5.00 hours for the enteric capsule, and 6.50–7.50 hours for the SR tablet formulations.

TBI-223 exposure (maximum concentration [*C*_max_] and area-under-the-curve values) increased in a nearly dose-proportional manner with an increase in dose under fasted conditions over the 50–2,600 mg dose range of the oral suspension administered in the fasted state. Slope estimates and 90% confidence intervals (90% CI) were 0.91 (0.86–0.96) for *C*_max_, 1.13 (1.08–1.18) for AUC from time zero to the time of the last quantifiable concentration (AUC_0-*t*_), and 1.13 (1.08–1.18) for AUC from time zero extrapolated to infinity (AUC_0-inf_) (Table S2; Fig. S2 to S4).

Food had little effect on the oral suspension at 1,200 mg, with geometric mean ratios, fed/fasted, of *C*_max_, AUC_0-*t*_, and AUC_0-inf_ of 1.03 (Table S3). However, for the 2,000 mg IR tablet formulation, the ratio was 1.51 (95% CI 1.28–1.79) for *C*_max_ and 1.16 (95% CI 1.12–1.19) for both AUCs (Table S4). The SR formulations were administered only in the fed state in CL-001.

After the administration of the enteric capsule, TBI-223 *C*_max_ was 42% that of the oral suspension, but AUCs of the capsule were 94%–95% that of the suspension (Table S5). This outcome demonstrated uptake in the duodenum and, therefore, supported evaluating SR formulations that were introduced in CL-001 and carried forward to CL-002. Under fed conditions, the mean *C*_max_ for the SR tablets was less than half that of the IR tablet, while the mean AUC_0-inf_ was approximately 70%–80% (Table 4).

#### CL-002

**Fig 2:**
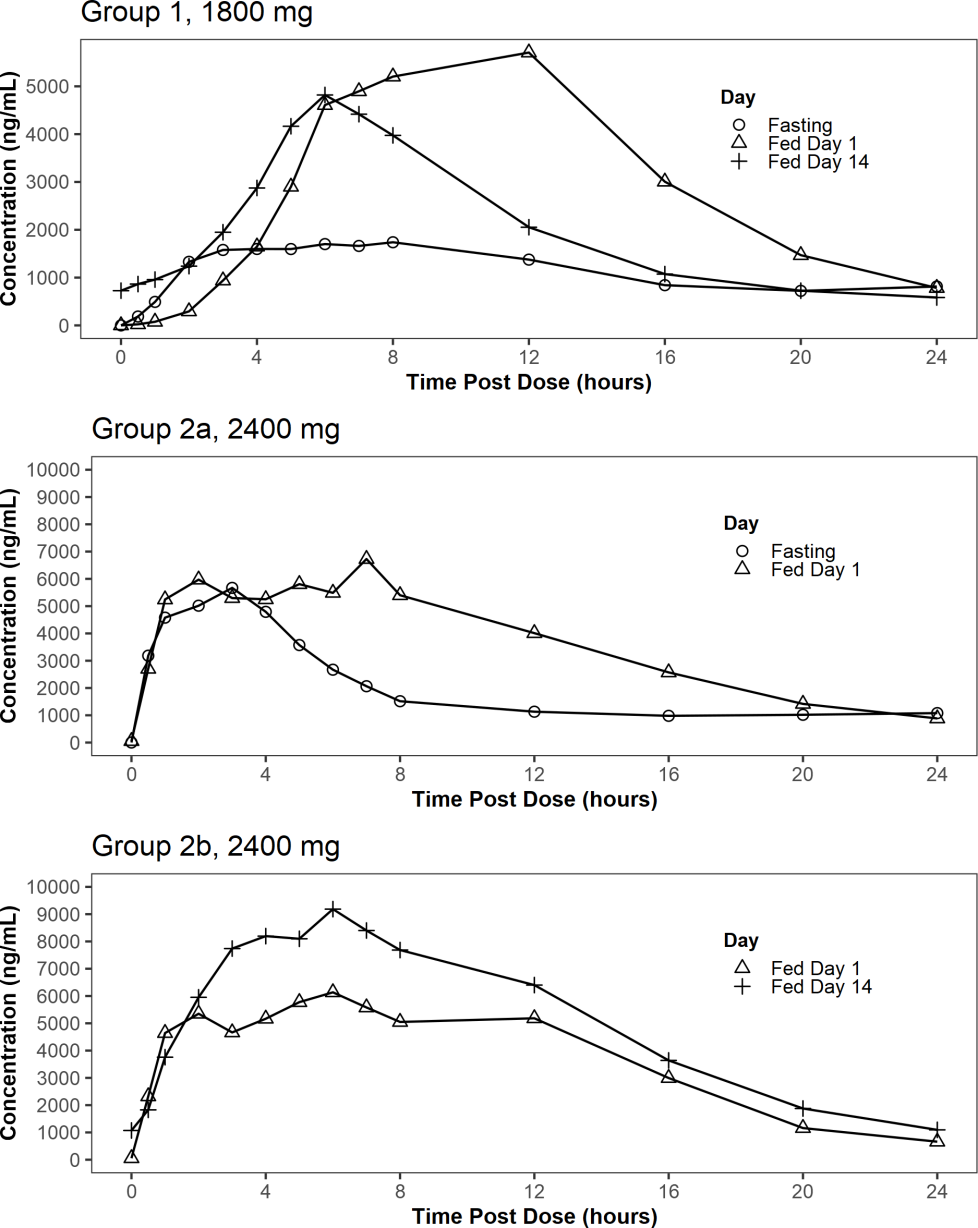
Mean plasma concentration-time profiles of TBI-223 after single and multiple doses of TBI-223 in MAD Study CL-002 by treatment, group, and day.

The mean concentration profiles of TBI-223 are shown in Fig. 2. PK parameters are summarized in Table 5. For M2, see Fig. S5 and Table S6.

**TABLE 5:** TBI-223 exposure metrics in MAD study CL-002^*f*^

Group		*T*_max_ (h)	*C*_max_ (μg/mL)	AUC_0-24_ (μg·h/mL)	AUC_0-inf_ (μg·h/mL)	*T*_1/2_ (h)	CL/F or CL_ss_/F (L/h)	*V*_*z*_/F or V_ZSS_/ F (L)
	*n*	Median (range)	Mean (SD)	Mean (SD)	Mean (SD)	Mean (SD)	Mean (SD)	Mean (SD)
Fasting								
1: 1,800 mg	9	5.0 (3.0,30.0)	2.58 (2.12)	27.7 (16.3)	37.9 (19.7)	3.36 (1.74)	86.6 (56.2)	345 (139)
2a: 2,400 mg	9	2.0 (1.0,3.0)	6.92 (3.15)	47.3 (18.9)	62.3 (26.1)^*a*^	4.93 (2.26)^*a*^	47.4 (25.2)^*a*^	327 (210)^*a*^
1st fed day								
1: 1,800 mg	9	12.0 (6.0,16.0)	7.26 (2.63)	70.6 (29.4)	76.5 (30.5)	4.50 (2.06)	34.8 (10.3)	229 (139)
2a: 2,400 mg	9	5.0 (0.5,16.0)	8.79 (2.72)	87.1 (33.8)	111 (30.1)^*b*^	2.93 (0.63)^*b*^	22.9 (5.6)^*b*^	99 (40)^*b*^
2b: 2,400 mg	9	5.0 (0.5,16.0)	8.88 (2.81)	88.6 (31.0)	101 (29.5)^*c*^	3.10 (0.80)^*c*^	25.5 (7.1)^*c*^	116 (47)^*c*^
14th fed day								
1: 1,800 mg	9	5.0 (4.0, 8.0)	5.10 (3.29)	47.3 (29.0)	NA^*e*^	7.30 (7.07)	74.8 (53.9)	701 (669)
2b: 2,400 mg	9	6.0 (2.0, 12.0)	11.4 (3.25)	121 (44.4)	NA^*e*^	4.56 (1.31)^*d*^	21.9 (6.5)	136 (51)^*d*^

^*a*^ *n* = 8 (*n* < 9 because tail slope could not be estimated).

^*b*^ *n* = 5 (*n* < 9 because tail slope could not be estimated).

^*c*^ *n* = 7 (*n* < 9 because tail slope could not be estimated).

^*d*^ *n* = 8 (*n* < 9 because tail slope could not be estimated).

^*e*^ NA, not relevant after multiple dosing.

^*f*^ 1,800 mg as 3× SR1 600 mg tablets; 2,400 mg as 3× SR1 600 mg tablets + 1× IR 600 mg tablet. *T*_max_: time of the maximum plasma concentration. *C*_max_: maximum concentration. AUC_0-*t*_: area under the plasma concentration-time curve from time zero to the time of the last quantifiable concentration, as calculated by the linear trapezoidal rule. AUC_0-inf_: area under the plasma concentration-time curve from the time of dosing extrapolated to infinity. *T*_1/2_: terminal elimination half-life. CL/F: apparent total plasma clearance after an oral dose. *V*_*z*_/F: apparent volume of distribution after an oral dose.

Food increased exposure of TBI-223 after single doses of the SR formulation two- to threefold. Geometric mean ratios of *C*_max_, AUC_0-24_, and AUC_inf_ were 3.23, 2.73, and 2.21, respectively, in group 1. In group 2a, which received a combination of SR and IR tablets, the corresponding ratios were 1.29, 1.85, and 1.37 (Table S7). For M2, see Table S8.

Over 14 days of dosing 2,400 mg in the fed state in group 2b, TBI-223 *C*_max_ increased from 8.88 to 11.4 µg/mL and AUC_0-24_ from 88.6 to 121 µg·h/mL. However, in group 1, where the dose was 1,800 mg, *C*_max_ decreased from 7.26 to 5.10 µg/mL and AUC_0-24_ from 70.6 to 47.3 µg·h/mL (Table 5; see Tables S9 and S10 for accumulation ratios). The behavior in group 1 may have been the result of alternating breakfast menus, with different menus on the odd-numbered 1st fed day and the even-numbered 14th fed day. The 14th fed day’s *C*_max_ was still larger than the value on the fasted day (5.10 vs 2.58 µg/mL), and similarly for AUC_0-24_ (47.3 vs 27.7 µg·h/mL) (Table 5). For both dose groups, assessment of trough concentrations indicated that steady state was achieved by the seventh fed dose.

### Safety

### CL-001 and CL-002 safety summary

In both CL-001 and CL-002, treatment with TBI-223 was generally safe and well tolerated by healthy participants. There were no fatal, serious, or severe treatment-emergent AEs (TEAEs).

TEAEs for CL-001 are listed by system organ class (SOC) and preferred term (PT) in Table 6. In CL-001, all TEAEs were mild in severity except for four participants who each experienced one TEAE of moderate severity (hypersensitivity, vomiting, urticaria, and increased bilirubin, respectively). Gastrointestinal AEs occurred at the highest doses of TBI-223 (2,000 and 2,600 mg) in 30% (6 out of 20) of participants but not in participants receiving placebo or lower doses of TBI-223. These TEAEs included nausea, vomiting, and abdominal pain. TEAEs in the neurological SOC occurred in a dose-dependent manner in 9 of 72 (12.5%) participants receiving TBI-223 but not in those receiving placebo. Of the 20 participants, 7 participants (35%) in the highest dose groups (2,000 and 2,600 mg) reported neurological TEAEs, including dizziness (25%, 5 out of 20 participants) and headache (10%, 2 out of 20 participants). Two participants receiving single doses of TBI-223 (one on 100 mg and the other on 2,600 mg) experienced clinically significant orthostatic tachycardia (99–140 bpm and 83–131 bpm), which resolved. No other clinically significant electrocardiogram (ECG) changes were noted.

**TABLE 6:** TEAEs in SAD study CL-001 by system organ class and preferred term^*a*^

Dose (mg)	Placebo	50–600	1,200–1,800	2,000–2,600	Any TBI-223
*N*	14	26	26	20	72
Participants with any TEAE	4 (28.6)	4 (15.4)	6 (23.1)	9 (45)	19 (26.4)
Blood and lymphatic system disorders	1 (7.1)	0 (0)	0 (0)	0 (0)	0 (0)
Neutropenia	1 (7.1)	0 (0)	0 (0)	0 (0)	0 (0)
Cardiac disorders	0 (0)	1 (3.8)	0 (0)	1 (5)	2 (2.8)
Tachycardia	0 (0)	1 (3.8)	0 (0)	1 (5)	2 (2.8)
Gastrointestinal disorders	0 (0)	0 (0)	0 (0)	6 (30)	6 (8.3)
Abdominal pain	0 (0)	0 (0)	0 (0)	1 (5)	1 (1.4)
Nausea	0 (0)	0 (0)	0 (0)	6 (30)	6 (8.3)
Vomiting	0 (0)	0 (0)	0 (0)	2 (10)	2 (2.8)
General disorders and administration site conditions	0 (0)	2 (7.7)	4 (15.4)	1 (5)	7 (9.7)
Chest discomfort	0 (0)	0 (0)	0 (0)	1 (5)	1 (1.4)
Energy increased	0 (0)	1 (3.8)	0 (0)	0 (0)	1 (1.4)
Medical device site reaction	0 (0)	1 (3.8)	0 (0)	0 (0)	1 (1.4)
Vessel puncture site inflammation	0 (0)	0 (0)	2 (7.7)	1 (5)	3 (4.2)
Vessel puncture site pain	0 (0)	0 (0)	1 (3.8)	0 (0)	1 (1.4)
Vessel puncture site reaction	0 (0)	0 (0)	1 (3.8)	0 (0)	1 (1.4)
Vessel puncture site swelling	0 (0)	0 (0)	1 (3.8)	0 (0)	1 (1.4)
Immune system disorders	1 (7.1)	0 (0)	0 (0)	0 (0)	0 (0)
Hypersensitivity	1 (7.1)	0 (0)	0 (0)	0 (0)	0 (0)
Investigations	0 (0)	1 (3.8)	0 (0)	0 (0)	1 (1.4)
Blood bilirubin increased	0 (0)	1 (3.8)	0 (0)	0 (0)	1 (1.4)
Musculoskeletal and connective tissue disorders	0 (0)	0 (0)	1 (3.8)	1 (5)	2 (2.8)
Back pain	0 (0)	0 (0)	1 (3.8)	1 (5)	2 (2.8)
Nervous system disorders	0 (0)	0 (0)	2 (7.7)	7 (35)	9 (12.5)
Disturbance in attention	0 (0)	0 (0)	0 (0)	1 (5)	1 (1.4)
Dizziness	0 (0)	0 (0)	1 (3.8)	5 (25)	6 (8.3)
Headache	0 (0)	0 (0)	1 (3.8)	2 (10)	3 (4.2)
Skin and subcutaneous tissue disorders	1 (7.1)	1 (3.8)	0 (0)	0 (0)	1 (1.4)
Skin discoloration	1 (7.1)	0 (0)	0 (0)	0 (0)	0 (0)
Urticaria	0 (0)	1 (3.8)	0 (0)	0 (0)	1 (1.4)
Vascular disorders	1 (7.1)	0 (0)	0 (0)	0 (0)	0 (0)
Hypotension	1 (7.1)	0 (0)	0 (0)	0 (0)	0 (0)

^*a*^ Number of participants (%).

The TEAEs for CL-002 are listed by SOC and PT in Table 7. All TEAEs were mild in severity except for two participants who experienced TEAEs of moderate severity (palpitations and increased ALT). More participants on TBI-223 than on placebo developed TEAEs in the investigations SOC (45% or 10 out of 22 participants, on TBI-223; 0 out of 6 participants on placebo). Of the 10 TEAEs, 8 in the Investigations SOC on TBI-223 were participants’ positive COVID-19 tests. In four participants, the COVID-19 infections occurred during treatment leading to early discontinuation of treatment and study withdrawal. The other four participants tested positive for COVID-19 during the follow-up period and completed the study. All COVID-19 infections were mild, resolved, and were not considered related to the study product. As in CL-001, more participants on TBI-223 (nfo14% or 3 out of 22 participants) had gastrointestinal disorders (including constipation, nausea, and dry mouth) than those on placebo (0 out of 6 participants). Musculoskeletal and connective tissue disorders (back pain and neck pain) were also more frequent on TBI-223 (14% or 3 out of 22 participants) than placebo (0 out of 6 participants), as were neurological disorders (9% or 2 out of 22 participants with headache on TBI-223 vs 0 out of 6 participants on placebo) and skin disorders (9% or 2 out of 22 participants on TBI-223 vs 0 out of 6 participants on placebo).

**TABLE 7:** TEAEs in MAD study CL-002 by system organ class and preferred term^*a*^

Dose (mg)	Placebo	1,800	2,400	Any TBI-223
*N*	6	9	13	22
Participants with any TEAE	2 (33)	5 (56)	10 (77)	15 (68)
Cardiac disorders	0	0	1 (7.7)	1 (4.5)
Palpitations	0	0	1(7.7)	1 (4.5)
Eye disorders	1 (17)	0	0	0
Lacrimation increased	1 (17)	0	0	0
Gastrointestinal disorders	0	1 (11)	2 (15)	3 (14)
Constipation	0	1 (11)	0	1 (4.5)
Dry mouth	0	0	1 (7.7)	1 (4.5)
Nausea	0	0	1 (7.7)	1 (4.5)
General disorders and administrative site conditions	1 (17)	2 (22)	1 (7.7)	3 (13.6)
Chest discomfort	0	1 (11)	1 (7.7)	2 (9.1)
Facial discomfort	1 (17)	0	0	0
Xerosis	0	1 (11)	0	1 (4.5)
Infections and infestations	0	0	2 (15)	2 (9.1)
Post-viral fatigue syndrome	0	0	2 (15)	2 (9.1)
Investigations	0	3 (33)	7 (54)	10 (45)
Alanine aminotransferase increased	0	1 (11)	0	1 (4.5)
Aspartate aminotransferase increased	0	1 (11)	0	1 (4.5)
Hemoglobin decreased	0	1 (11)	0	1 (4.5)
SARS-CoV-2 test positive	0	1 (11)	7 (54)	8 (36)
White blood cells increased	0	1 (11)	0	1 (4.5)
Musculoskeletal and connective tissue disorders	0	1 (11)	2 (15)	3 (14)
Back pain	0	1 (11)	0	1 (4.5)
Neck pain	0	0	2 (15)	2 (9.1)
Nervous system disorders	0	1 (11)	1 (7.7)	2 (9.1)
Headache	0	1 (11)	1 (7.7)	2 (9.1)
Skin and subcutaneous disorders	0	1 (11)	1 (7.7)	2 (9.1)
Pityriasis rosea	0	0	1 (7.7)	1 (4.5)
Urticaria	0	1 (11)	0	1 (4.5)

^*a*^ Number of participants (%).

### QTc analysis

Using data from the oral suspension-fasting groups in CL-001, modeling of the relationships between plasma concentrations and change-from-baseline in QTcF (ΔQTcF) found significant effects for both TBI-223 and M2 in separate models for each. The model equations for placebo-adjusted ΔQTcF, (ΔΔQTcF) were

ΔΔQTcF (ms) = 1.56 (ms) +0.00070 [ms/(ng/mL)] × TBI-223 concentration (ng/mL)

ΔΔQTcF (ms) = 1.47 (ms) +0.0028 [ms/(ng/mL)] × M2 concentration (ng/mL)

Figure S6 displays the data with the superimposed model-predicted line and confidence intervals for TBI-223. For a single dose of 1,200 mg TBI-223, the predicted effect on ΔΔQTcF at the geometric mean *C*_max_ of TBI-223 (8,136 ng/mL) was 7.28 ms (upper bound of 90% CI: 10.00) and at the geometric mean *C*_max_ of M2 (2,199 ng/mL) 7.68 ms (upper bound of 90% CI: 10.49). The concentration levels of TBI-223 and M2 at which the upper bound of two-sided 90% confidence intervals of the model-predicted ΔΔQTcF from the model with TBI-223 alone and the model with M2 alone crossed the 10 ms threshold were 8,142 and 2,045 ng/mL, respectively.

Similar results were found with the data from CL-002. The concentration levels of TBI-223 and M2 at which the 10 ms threshold was crossed were 7,468 and 1,827 ng/mL, respectively. These were close to the mean *C*_max_ on the first fed day at 1,800 mg for each analyte (Table 5; Table S6).

In CL-001, QTcF > 450 ms was observed in one subject each in the placebo, 600 mg, 1,200 mg fasted, 1,200 mg fed, SR1, and SR2 groups, and two participants in the 2,000 mg suspension group. No participants had QTcF > 480 ms. ΔQTcF > 30 ms was observed in one subject each in the placebo, 600 mg, 1,200 mg fed, 2,600 mg, and SR3 groups, two participants at 300 mg suspension, and three participants at 100 mg. No participants had ΔQTcF >60 ms.

In CL-002, one subject at 1,800 mg and one placebo subject had QTcF > 450 ms; none > 480 ms. Three participants at 1,800 mg, two at 2,400 mg, and three on placebo had ΔQTcF > 30 ms; none > 60 ms.

## DISCUSSION

TBI-223 is one of several novel oxazolidinones under development for TB (3, 16). A primary motivation is to find an agent that contributes as much sterilizing activity as linezolid to anti-TB regimens but without the toxicities associated with linezolid’s MPS inhibition.

Several PK observations merit discussion. (i) Food increased exposure for the tablet but not for the suspension. This may have been due to food increasing tablet disintegration and/or longer retention of the tablet in the upper gastrointestinal tract before emptying, allowing more absorption at the duodenal-jejunal junction. (ii) In CL-002, bioavailability seemed to vary with meal types that had similar carbohydrate, fat, and protein calories that met the FDA criteria for a high-fat, high-calorie breakfast. This may have been due to enhanced absorption with an egg-based meal due to egg lecithin (17) in the 2,400 mg group vs the sausage-based meal in the 1,800 mg group. The meal used for the food-effect assessments in CL-001 was more similar to the egg-based meal in CL-002 but not the same because the studies were conducted at different sites. The results of CL-002 indicate that the estimated effects of food in CL-001 must be interpreted with caution as different meals may have different effects. (iii) Although exposure increased approximately dose proportionally when TBI-223 was administered as single doses, and the half-life was only 2–4 hours, exposure of TBI-223 over 14 days of dosing in the 2,400 mg group with the constant meal type increased by 30%–36%. This may have been due to nonlinear clearance, as found by population pharmacokinetic modeling (18). Steady state was judged to have been reached by the seventh fed dose.

TBI-223 was generally safe and well tolerated at single doses up to 2,600 and 2,400 mg once daily for 14 days. There were no fatal, serious, or severe AEs, and no specific safety signals were identified. Four participants on TBI-223 discontinued treatment because of COVID-19, deemed unrelated to the study drug. There was no evidence of MPS-related toxicities, such as peripheral neuropathy and myelosuppression, although 14 days of exposure may be too short of a time frame to fully assess such risks. In the Nix-TB (8) study, the majority of linezolid dose modifications for neuropathy occurred after 3 months of treatment; dose modifications for myelosuppression were rarer and tended to occur within the first 2 months but not within the first 14 days. Similar results were seen in the ZeNix trial (9). The highest mean *C*_max_ and AUC for TBI-223 after 14 days of dosing, 11.4 µg/mL and 121 µg·h/mL (Table 5), are below the no-observed-adverse-effect levels (NOAELs) from 3-month rat and dog toxicity studies. Additionally, in the nonclinical toxicology studies, there was no histopathology evidence of nerve degeneration with TBI-223, which has been observed as peripheral neuropathy for linezolid and observed as optic nerve degeneration in rats in the nonclinical program as mentioned in the linezolid label (19).

Linezolid has been associated with prolonged QTc when co-administered with other QTc-prolonging drugs (20–23). As a potential replacement for linezolid, TBI-223 would likely also be combined with such drugs, such as bedaquiline and moxifloxacin. In the two studies considered here, concentration-QTc modeling found significant relationships of QTc with concentrations of both TBI-223 and its M2 metabolite. However, categorical evaluations of QTc prolongation found that TBI-223 did not differ markedly from placebo.

The primary limitation of the two studies described here is the insufficiency of 14 days’ dosing to clinically differentiate TBI-223 from linezolid with regard to MPS-related adverse events. A second limitation is that the lack of accumulation in the 1,800 mg group of the MAD study was not definitively explained, although its cause was conjectured to be the alternation of different breakfast menus. Whether this leads to exaggerated PK variability in outpatient settings and whether that matters for efficacy or safety remains to be seen in future studies. It renders dose selection based on target exposure riskier. Nonetheless, an attempt at clinical dose optimization using translational modeling has been undertaken (18).

Overall, the results of the two studies considered here support the continued investigation of TBI-223 as an anti-TB agent. The next such investigation will be A5409/RAD-TB (NCT06192160), a phase 2 randomized, controlled, open-label, dose-ranging, platform protocol to evaluate the safety and efficacy of multidrug regimens for the treatment of adults with drug-susceptible pulmonary TB.

## MATERIALS AND METHODS

### Study design

CL-001 was conducted at a single site in San Antonio, TX, USA, and CL-002 at a single site in Fair Lawn, NJ, USA, under the sponsorship of TB Alliance.

#### CL-001

CL-001 started as a study to evaluate the safety, tolerability, and PK of single ascending doses of TBI-223 oral suspension. When the short half-life of TBI-223 was discovered, an arm was added to evaluate intestinal absorption with an enteric-capsule formulation, and then arms were added to evaluate SR and IR tablet formulations. Reporting groups are summarized in Table 1. An arm comprised one of the reporting groups 1–9b together with matching placebos from reporting group 0. All were parallel arms, except for some crossovers from suspension to enteric capsule (groups 3a to 3b) and fasting to fed (groups 5a to 5b and 9a to 9b), as indicated in Table 1. In each of the seven oral suspension arms, participants were randomized as six to active and two to placebo, except for the food-effect cohort, where eight were randomized to active and two to placebo. Participants experiencing emesis could be replaced if the emesis could potentially impact drug absorption and, therefore, the pharmacokinetic data.

The starting dose in CL-001 and subsequent dose escalations were guided by safety margins relative to toxicology studies, consistent with the International Conference on Harmonisation M3(R2) guideline (Nonclinical safety studies for the conduct of human clinical trials for pharmaceuticals—Scientific guideline), and by emerging PK and safety data during the study. A starting dose of 50 mg was selected based on the human equivalent dose of the most sensitive nonclinical species’ NOAEL, providing a 32-fold safety margin (internal data). The initially planned maximum dose was 1,400 mg, but following protocol-specified procedures for decision-making, including IRB approval, the maximum dose eventually reached 2,600 mg.

Participants were housed in the clinic from at least 48 hours prior until 7 days after dosing. Day −1 and Day 1 were the day before and the day of dosing, respectively. In the first, 50 mg arm, a sentinel subset of three participants (two active and one placebo) was dosed at least 24 hours before the remaining five participants (four active and one placebo). The remaining oral suspension arms except for the newly enrolled participants in reporting group 3b were dosed in two subsets of four participants each (three active and one placebo) at least 24 hours apart.

For the two food-effect cohorts, the breakfast prior to the fed dose consisted of three eggs fried in butter, yolks broken; 4 ounces of hash brown potatoes; two slices of white bread toast; two pats of butter; and 8 ounces of whole milk.

#### CL-002

CL-002 was a partially blinded, placebo-controlled, randomized MAD study. The once-daily dose formulations in this study were selected to achieve high AUC and TMIC by combining SR tablets (SR1, one of the three formulations tested in CL-001) and IR tablets. Three dose levels were originally planned: 1,800 mg (3 × 600 mg SR1), 2,400 mg (3 × 600 mg SR1 + 1× 600 mg IR), and 3,000 mg (4 × 600 mg SR1 + 1× 600 mg IR). PK modeling predicted that these doses would keep *C*_max_ below the preclinical NOAEL of 20.2 µg/mL and that the highest dose would yield AUC_0-24h_ of 115 µg·h/mL, near a target of 124 µg·h/mL, which was found in a dose-fractionation study in BALB/c mice to yield a 2-log reduction in lung colony-forming units (internal data). When the predicted AUC_0-24_ for 3,000 mg was surpassed by 2,400 mg, the 3,000 mg dose level was canceled, as permitted by the protocol.

Both dose groups enrolled 12 participants, with 9 randomized to active and 3 randomized to matching placebo formulations. Dosing began on Day 1 under fasted conditions, followed by a 3-day washout period, and then by multiple doses administered after a high-calorie, high-fat meal from Day 4 to Day 17 (total of 14 days).

In the 2,400 mg group, four participants randomized to TBI-223 were discontinued due to COVID-19 infection; they received at least the Day 1 (fasted) dose and were discontinued during the 14-day dosing-under-fed conditions. Four replacement participants were enrolled, who began dosing on Day 1 and continued through Day 14 (14 days of dosing), with all doses administered after a high-calorie, high-fat meal; i.e., the replacement participants did not receive an initial dose under fasted conditions, because sufficient data on fasted-vs-fed had been provided by the original participants. Reporting groups are summarized in Table 2.

For the 1,800 mg group, during fed Days 4–17, the high-calorie, high-fat meal alternated between two different menus: (i) two eggs fried in butter, two strips of bacon, two slices of toast with butter, 4 ounces of hash browns, and 8 ounces of whole milk; and (ii) three sausage patties, one cup of Cheerios, two slices of French toast, and 10 ounces of whole milk. Menu #1 was used on odd-numbered days, and menu #2 was used on even-numbered days. Both meals contained approximately 150 protein calories, 250 carbohydrate calories, and 500–600 fat calories. For the 2,400 mg group, only menu #1 was used for all 14 fed days.

#### Exclusion criteria

For CL-001, participants were excluded if they had a history of clinically significant disease or condition; an abnormal neurological examination; abnormal serum magnesium, potassium, or calcium laboratory values, or positive results at screening for human immunodeficiency virus, hepatitis B surface antigen, or hepatitis C antibodies; alcohol and/or substance abuse within the past 2 years or a positive pre-dosing urine screen for drug/alcohol/cotinine; sensitivity to linezolid or sulfa drugs; a finding of clinically significant ECG abnormalities or a finding of abnormal pulse or blood pressure on repeated testing at screening; a QTcF (QT interval corrected for heart rate by Fridericia’s formula) interval of >450 ms for males or >470 ms for females at screening, on Day −1, or pre-dose on Day 1, or a history of prolonged QT syndrome; family history of prolonged QT syndrome or unexplained sudden death; exposure to medications known to cause ECG abnormalities and/or QTc prolongation; exposure to prescription medications within 14 days, over-the-counter medications except acetaminophen, herbal medications, or vitamin supplements within 7 days; and pregnancy or lactation. Participants were also excluded if they had used any significant inhibitors of CYP enzymes and/or significant inhibitors or substrates of P-gp and/or organic anion transporting polypeptides within 14 days, and any inducers of CYP enzymes and/or P-gp, including St. John’s Wort, within 30 days prior to the first dose of the study drug.

For CL-002, the exclusion criteria were similar to CL-001.

### Assessments

#### Safety

Safety was assessed throughout both studies for all participants. Safety assessments included physical and neurological examinations, vital signs, 12-lead ECGs, cardiac monitoring, AEs, and clinical laboratory tests (including hematology, serum chemistry, coagulation, and urinalysis). All AEs were collected from the time of screening but only treatment-emergent AEs, which occurred after dosing, are reported in this manuscript. Female participants had blood collected for serum pregnancy testing. Postmenopausal females had blood collected to measure follicle-stimulating hormone levels.

#### ECGs

In CL-001 groups receiving the oral suspension, serial ECGs were extracted from Holter recordings at three time points before dosing on Day 1 (−45, –30, and −15 minutes) and at 0.5, 1, 2, 3, 4, 5, 6, 8, 12, 16, 20, 24, 30, 36, and 48 hours post-dose. ECGs were also taken on Day −1 at time points corresponding to the pre-dose through 20-hour timepoints after dosing.

In CL-002 groups 0, 1, and 2a, 12-lead ECGs were obtained in triplicates at screening, Day −2, Day −1, and on Day 26 or upon early withdrawal. At all other time points, single ECGs were recorded. ECGs were recorded within 2 hours prior to dosing and at 3, 6, 12, and 16 hours post-dose on Days 1, 4, 8, 10, 12, 14, 17, and at unspecified times on Days 18 and 20, the check-out day for the original participants who completed the study.

For the replacement participants in group 2b, ECGs were obtained in triplicates at screening, Day −2, Day −1, and on Day 23 or upon early withdrawal. All other time points were single ECG readings. ECGs were done within 2 hours prior to time of dosing and at 3, 6, 12, and 16 hours post-dose on Days 1, 2, 3, 7, and 14, and at an unspecified time on Day 17, the check-out day for the four replacement participants.

### Blood sampling

#### CL-001

PK samples were drawn at pre-dose (0 h) and at 0.5, 1.0, 1.5, 2, 3, 4, 5, 6, 7, 8, 12, 16, 20, 24, 30, 36, 42, 48, and 72 hours after administration of the investigational product.

#### CL-002

PK samples were collected pre-dose on each dosing day and post-dose at the following time points:

- Day 1: 0.5, 1, 2, 3, 4, 5, 6, 7, 8, 12, 16, 20, and 24 hours and, except for the four replacement participants, 30, 36, 42, 48, 54, 60, and 72 hours. The 72-hour sample was the pre-dose sample of Day 4.
- Day 4, except for the four replacement participants: 0.5, 1, 2, 3, 4, 5, 6, 7, 8, 12, 16, 20, and 24 hours. The 24-hour sample was the pre-dose sample of Day 5.
- Day 17 or, for the four replacement participants, Day 14: 0.5, 1, 2, 3, 4, 5, 6, 7, 8, 12, 16, 20, 24, 30, 36, 42, 48, 54, 60, and 72 hours.

### Bioanalytical procedures

Samples were collected in appropriately labeled, pre-chilled 4.0 mL evacuated tubes containing K_3_-EDTA and immediately placed on wet ice, then processed to plasma within 60 minutes. Blood samples were centrifuged at 1,500 *g* for 10 minutes at approximately 4°C, and the resulting plasma was transferred into two appropriately labeled 5.0 mL polypropylene vials, the first containing at least 0.5 mL, and the second containing the remainder of the plasma. Within 90 minutes of collection, samples were frozen at approximately −20°C ± 10°C and remained in the freezer pending shipment to Alliance Pharma Inc. for analysis. Samples were analyzed during the study to support dose-escalation decisions.

Plasma concentrations of TBI-223 were determined using a validated liquid chromatography-tandem mass spectrometry method (unpublished). The lower limit of quantification was 5.00 ng/mL for both TBI-223 and M2.

Standard noncompartmental PK parameters were calculated for TBI-223 and its M2 metabolite using Phoenix WinNonlin (version 8.1, Certara, L.P.) for CL-001 and SAS (version 9.4, SAS Institute, Inc.) for CL-001 and CL-002.

### Statistical analysis

Statistical assessments were performed on TBI-223 and M2 using SAS (Version 9.4, SAS Institute, Inc.).

#### CL-001: dose proportionality

Dose proportionality was assessed for the following PK parameters of TBI-223: *C*_max_, AUC_0-*t*_, and AUC_inf_. Statistical analyses were performed using a power model (24) of the following general form:

ln(PK) = ln(β_0_) + *β*_1_·ln(dose) + ϵ,

where PK is the pharmacokinetic parameter tested (e.g., *C*_max_ or AUC), ln(β_0_) is the *y*-intercept, *β*_1_ is the slope, and ϵ is an error term.

The estimate of *β*_1_ with the 90% CIs (24) was reported, along with the associated *P* value. A *β*_1_ value of approximately 1 indicates linearity. Dose-proportionality plots were also created.

#### CL-001: food effect and enteric capsule vs oral suspension

Statistical comparison of the PK parameters of exposure (*C*_max_, AUC_0-*t*_, and AUC_0-inf_) was performed using an analysis of variance (ANOVA) model for a two-way crossover design on the ln-transformed data with treatment as the fixed effects and subject as a random effect. Conclusions regarding the results of the statistical analysis of PK parameters across treatments were based on the ratio of the geometric means expressed as a percentage (100 × Fed/Fasted or 100 × Capsule/Suspension) and the 90% CI about the ratio.

#### CL-002: food effect

Statistical comparison of the PK parameters of exposure (*C*_max_, AUC_0-24_, and AUC_0-inf_) was performed using an ANOVA model on the ln-transformed data from each dose group separately, with effects for Day 1 vs Day 4 and subject. Conclusions regarding the results of the statistical analysis were based on the ratio of the geometric means expressed as a percentage (100 × Fed/Fasted) and the 90% CI about the ratio. The food effect on *T*_max_ was assessed using the Wilcoxon signed-rank test.

#### CL-002: steady-state attainment

Achievement of steady state was assessed through analysis of trough concentrations from the 7th to 14th days of fed dosing, using analysis of variance with effects of subject and day (taking day as a 1-degree-of-freedom regressor effect). If the effects of day and subject-day interaction were not found to be significant at the 5% level, then steady state was judged to have been achieved.

### Concentration-QTc analysis

The relationship between ΔQTcF and concentrations of TBI-223 and M2 was evaluated for CL-001 and CL-002 separately using linear mixed-effects models, with covariates comprising plasma concentrations of TBI-223 and M2 together or separately (0 for placebo), treatment (active vs placebo), time point, and centered baseline QTcF (i.e., baseline QTcF for individual subject subtracting the population mean baseline QTcF for all participants). Random intercept and slope per subject were included, as supported by the model. Analyses were done by ERT (now Clario, https://clario.com/) for CL-001 and by Cardiac Safety Consultants Ltd. (https://cardiacsafetyconsultants.com/) for CL-002.

## SUPPLEMENTAL MATERIAL

Supplemental material aac.01542-24-s0001.pdf Tables S1 to S10; Figures S1 to S6.

ASM does not own the copyrights to Supplemental Material that may be linked to, or accessed through, an article. The authors have granted ASM a non-exclusive, world-wide license to publish the Supplemental Material files. Please contact the corresponding author directly for reuse.
