# Supplementary material for: Pharmacokinetics, tolerability, and safety of TBI-223, a novel oxazolidinone, in healthy participants
Source: Antimicrob Agents Chemother. 2025 Mar 11;69(4):e01542-24. doi: 10.1128/aac.01542-24 (PMC11963608; doi:10.1128/aac.01542-24)
Supplement: Supplemental material — Tables S1 to S10; Figures S1 to S6. [file aac.01542-24-s0001.pdf]

Pharmacokinetics, Tolerability, and Safety of TBI-223, a Novel Oxazolidinone, in Healthy Participants: Supplemental Material

Antonio Lombardi[a,#], Fran Pappas[a], Paul Bruinenberg[b] Jerry Nedelman[a], Rajneesh Taneja[a], Dean Hickman[a], Maria Beumont[a], Eugene Sun[a]

[a]Global Alliance for TB Drug Development, New York, New York, USA

[b]Vast Therapeutics, Morrisville, North Carolina, USA

Keywords: TBI-223, oxazolidinone, tuberculosis, pharmacokinetics, phase 1, antimicrobial safety

Running Title: TBI-223 FIH in Healthy Adults

#Address correspondence to antonio.lombardi-consultant@tballiance.org

Supplemental Tables

Supplemental Table S1. TBI-223 M2 Exposure Metrics in CL-001 SAD Study

Group		T_{max} (h)	C_{max} (μg/mL)	AUC_{0-t} (μg.h/mL)	AUC_{0-inf} (μg.h/mL)	$T_{1/2}$ (h)
	n	Median (range)	Mean (SD)	Mean (SD)	Mean (SD)	Mean (SD)
Suspension and Enteric Capsule						
50 mg susp, fasted	6	1.00 (0.500-3.00)	0.109 (0.0157)	0.633 (0.108)	0.663 (0.106)	2.62 (0.256)
100 mg susp, fasted	6	1.50 (1.00-3.00)	0.196 (0.0313)	1.05 (0.177)	1.08 (0.178)	2.41 (0.238)
300 mg susp, fasted	6	1.50 (1.50-3.00)	0.897 (0.195)	4.61 (0.885)	4.64 (0.881)	2.25 (0.403)
300 mg enteric, fasted	6	5.50 (3.00-16.0)	0.489 (0.137)	3.98 (0.516)	4.02 (0.511)	3.93 (1.36)
600 mg susp, fasted	6	2.50 (1.00-4.00)	1.41 (0.397)	8.85 (2.02)	8.89 (2.02)	3.17 (0.585)
1200 mg susp, fasted	8	2.00 (3.00-4.00)	2.24 (0.345)	16.4 (2.84)	15.9 (2.66)	3.33 (0.635)
1200 mg susp, fed	7	2.00 (3.00-4.00)	2.21(0.399)	15.8 (2.59)	15.8 (2.58)	2.72 (0.207)
2000 mg susp, fasted	6	3.50 (3.00-4.03)	3.55 (0.871)	32.8 (7.34)	32.9 (7.34)	4.44 (0.698)
2600 mg susp, fasted	6	4.00 (3.00-5.00)	3.65 (0.772)	36.6 (4.79)	36.7 (4.80)	3.74 (0.752)
Tablets						
1800 mg SR1, fed	6	7.50 (7.00-16.0)	1.83 (0.557)	22.5 (5.42)	22.7 (5.35)	4.21 (2.45)
1800 mg SR2, fed	6	7.50 (4.00-8.00)	2.43 (0.657)	22.6 (4.77)	22.7 (4.77)	2.77 (0.728)
1800 mg SR3, fed	6	8.00 (5.00-20.0)	2.48 (0.499)	28.3 (7.23)	28.4 (7.24)	3.61 (0.843)
2000 mg IR tab, fasted	6	3.00 (3.00-4.00)	2.77 (0.499)	25.1 (3.80)	25.1 (3.79)	3.24 (0.604)
2000 mg IR tab, fed	6	3.00 (1.50-4.00)	3.62 (0.847)	26.0 (3.53)	26.0 (3.54)	2.97(0.325)

Note: C_{max} and AUCs for SR-1, SR-2, and SR-3 were normalized to the 2000 mg dose; e.g., reported C_{max} = observed C_{max} × (2000/1800). Curves in Figure S2 are not so normalized. Exposure metrics from two participants at 2600 mg suspension, fasted, were excluded from summary statistics because of early emesis.

susp: suspension. SR: Sustained Release. IR: Immediate Release.

T_{max}: Time of the maximum plasma concentration. C_{max}: Maximum concentration. AUC_{0-t}: Area under the plasma concentration-time curve from time-zero to the time of the last quantifiable concentration, as calculated by the linear trapezoidal rule. AUC_{0-inf}: Area under the plasma concentration-time- curve from the time of dosing extrapolated to infinity. $T_{1/2}$: Terminal elimination half-life. CL/F: Apparent total plasma clearance after an oral dose. V_z/F: Apparent volume of distribution after an oral dose.

Supplemental Table S2. Assessment of Dose Proportionality Following Single Dose Administrations of TBI 223 under Fasted Conditions, Oral Suspension Groups 1 – 7.

Dependent Variable	Model Variable	Estimate (β_1)	Lower CI[a]	Upper CI[a]	Rho1[b]
$\ln(C_{max})$	ln(Dose)	0.9090	0.8576	0.9603	7.5391
$\ln(AUC_{0\text{-}t})$	ln(Dose)	1.1314	1.0837	1.1791	4.9822
$\ln(AUC_{0\text{-}inf})$	ln(Dose)	1.1270	1.0785	1.1755	5.1521

Power Model: $\ln(PK)=\ln(\beta_0)+\beta_1*\ln(Dose)+e$, where PK is the pharmacokinetic parameter tested, $\ln(\beta_0)$ is the y-intercept, β_1 is the slope, and e is an error term
[a] 90% confidence intervals (Lower and Upper)
[b] Maximal dose ratio for proportionality. Rho1 was calculated as: $Rho1=\theta_H**(1/max(1-lower, upper-1))$, in which $\theta_H=1.33$.

Supplemental Table S3. Statistical Analysis of the Natural Log-Transformed Systemic Exposure Parameters of TBI 223 after a Single 1200 mg Dose of TBI-223 Oral Suspension under Fed (Test) and Fasted (Reference) Conditions

Dependent Variable	Geometric Mean[a] Test	Geometric Mean[a] Ref	Ratio (%)[b] (Test/Ref)	90% CI[c] Lower	90% CI[c] Upper	Power	ANOVA CV%
**C_{max}**	8760	8470	103.34	87.00	122.75	0.7093	17.01
**$AUC_{0\text{-}t}$**	45900	44500	103.23	97.80	108.96	0.9996	5.21
**$AUC_{0\text{-}inf}$**	45900	44700	102.79	96.37	109.64	0.9980	5.57

[a] Geometric Mean based on Least Squares Mean for TBI-223 administered under fed (Test) and Fasted (Reference) conditions
[b] Ratio (%) = Geometric Mean (Test)/Geometric Mean (Ref)
[c] 90% Confidence Interval

Supplemental Table S4. Statistical Analysis of the Natural Log-Transformed Systemic Exposure Parameters of TBI-223 after a Single 2000 mg Dose (2 x 1000 mg) of TBI-223 IR Tablets under Fed (Test) and Fasted (Reference) Conditions

Dependent Variable	Geometric Mean[a]		Ratio (%)[b]	90% CI[c]		Power	ANOVA
	Test	Ref	(Test/Ref)	Lower	Upper		CV%
C_{max}	19100	12600	151.24	127.91	178.82	0.7360	14.48
AUC_{0-t}	97600	84300	115.73	112.08	119.50	1.0000	2.76
AUC_{0-inf}	97600	84400	115.70	112.06	119.47	1.0000	2.75

[a] Geometric Mean based on Least Squares Mean for TBI-223 IR tablets administered under fed (Test) and fasted (Ref) conditions
[b] Ratio(%) = Geometric Mean (Test)/Geometric Mean (Ref)
[c] 90% Confidence Interval
IR: Immediate release

Supplemental Table S5. Statistical Analysis of the Natural Log-Transformed Systemic Exposure Parameters of TBI-223 after a Single 300 mg Dose of TBI-223 Enteric Capsule Formulation (Group 3b) and a Single 300 mg Dose of TBI-223 Oral Suspension (Group 3a)

Dependent Variable	Geometric Mean[a]		Ratio (%)[b]	90% CI[c]		Power	ANOVA
	Test	Ref	(Test/Ref)	Lower	Upper		CV%
C_{max}	1300	3070	42.46	31.57	57.10	0.3139	19.19
AUC_{0-t}	9300	9840	94.43	90.28	98.76	0.9991	2.71
AUC_{0-inf}	9350	9870	94.73	90.58	99.06	0.9991	2.70

[a] Geometric Mean based on Least Squares Mean for TBI-223 administered as a capsule (Test) and as an oral suspension (Reference) conditions
[b] Ratio(%) = Geometric Mean (Test)/Geometric Mean (Ref)
[c] 90% Confidence Interval

Supplemental Table S6: TBI-223-M2 Exposure Metrics in MAD Study CL-002

Group		T_{max} (h)	C_{max} (µg/mL)	AUC_{0-24} (µg.h/mL)	AUC_{0-inf} (h*µg/mL)	$T_{1/2}$ (h)
	n	Median (range)	Mean (SD)	Mean (SD)	Mean (SD)	Mean (SD)
Fasting						
1. 1800 mg	9	6.0 (3.0, 30.0)	0.717 (0.452)	9.6 (5.1)	13.7 (7.0)	4.15 (1.95)
2a. 2400 mg	9	3.0 (2.0,4.0)	1.71 (0.557)	16.1 (6.0)	21.4 (8.9)[a]	5.30 (1.94)[a]
1st Fed Day						
1. 1800 mg	9	12.0 (7.0,16.0)	1.88 (0.487)	20.6 (4.3)	23.4 (5.3)	4.94 (1.99)
2a. 2400 mg	9	7.0 (0.5,16.0)	2.23 (0.632)	26.7 (10.1)	28.6 (12.0)[b]	3.97 (0.71)[b]
2b. 2400 mg	9	7.0 (0.5, 16.0)	2.21 (0.538)	28.6 (8.3)	30.2 (9.2)[c]	4.01 (0.89)[c]
14th Fed Day						
1. 1800 mg	9	7.0 (4.0, 8.0)	1.25 (0.819)	14.8 (8.9)	NA[e]	8.75 (7.99)
2b. 2400 mg	9	7.0 (4.0, 12.0)	2.28 (0.560)	30.6 (9.3)	NA[e]	5.91 (1.6)[d]

n < 9 because tail slope could not be estimated: [a]n = 8. [b]n = 7. [c]n = 8. [d]n = 8.

[e]Not relevant after multiple dosing.

Supplemental Table S7. TBI-223 Food Effect, MAD Study CL-002

	TBI-223 1800 mg (N=9)	TBI-223 2400 mg (N=9)
**C_{max} (µg/mL)**		
Fasting Geometric Mean (SEM)	2.13 (1.14)	6.48 (1.10)
Fed Geometric Mean (SEM)	6.88 (1.14)	8.35 (1.10)
Ratio of Geometric Means	3.23	1.29
90% Confidence Interval	[2.29, 4.56]	[1.01, 1.65]
**AUC_{0-24} (µg.h/mL)**		
Fasting Geometric Mean (SEM)	24.3 (1.14)	44.1 (1.06)
Fed Geometric Mean (SEM)	66.5 (1.14)	81.5 (1.06)
Ratio of Geometric Means	2.73	1.85
90% Confidence Interval	[1.94, 3.84]	[1.58, 2.16]
**AUC_{0-inf} (µg.h/mL)**		
Fasting Geometric Mean (SEM)	32.8 (1.18)	57.4 (1.04)
Fed Geometric Mean (SEM)	72.3 (1.18)	78.4 (1.06)
Ratio of Geometric Means	2.21	1.37
90% Confidence Interval	[1.43, 3.40]	[1.15, 1.63]
**T_{max} (h)**		
Fasting Median	5.0	2.0
Fed Median	12.0	5.0
P value (Wilcoxon Signed Rank test, Fed vs. Fasting)	0.160	0.016

Supplemental Table S8. TBI-223-M2 Food Effect, MAD Study CL-002

	TBI-223 1800 mg (N=9)	TBI-223 2400 mg (N=9)
**Cmax (µg/mL)**		
Fasting Geometric Mean(SEM)	0.64 (1.12)	1.64 (1.06)
Fed Geometric Mean (SEM)	1.83 (1.12)	2.14 (1.06)
Ratio of Geometric Means	2.87	1.31
90% Confidence Interval	[2.14, 3.85]	[1.12, 1.52]
**AUC_{0-24} (µg.h/mL)**		
Fasting Geometric Mean (SEM)	8.7 (1.12)	15.1 (1.05)
Fed Geometric Mean (SEM)	20.2 (1.12)	24.9 (1.05)
Ratio of Geometric Means	2.33	1.65
90% Confidence Interval	[1.73, 3.13]	[1.44, 1.88]
**AUC_{0-inf} (µg.h/mL)**		
Fasting Geometric Mean (SEM)	11.9 (1.16)	19.8 (1.07)
Fed Geometric Mean (SEM)	22.8 (1.16)	23.7 (1.08)
Ratio of Geometric Means	1.92	1.19
90% Confidence Interval	[1.29, 2.85]	[0.96, 1.48]
**Tmax (h)**		
Fasting Median	6.0	3.0
Fed Median	12.0	7.0
P value (Wilcoxon Signed Rank test, Fed vs. Fasting)	0.711	0.008

Supplemental Table S9: TBI-223 Accumulation Ratios, MAD Study CL-002

Group	n	Geometric Mean Ratio (90% Confidence Interval)	
		C_{max}	AUC_{0-24}
1800 mg	9	0.630 (0.432, 0.918)	0.593 (0.367, 0.957)
2400 mg	9	1.30 (1.03, 1.64)	1.36 (1.11, 1.67)

Supplemental Table S10: TBI-223 M2 Accumulation Ratios, MAD Study CL-002

Group	n	Geometric Mean Ratio (90% Confidence Interval)	
		C_{max}	AUC_{0-24}
1800 mg	9	0.583 (0.409, 0.830)	0.621 (0.412, 0.934)
2400 mg	9	1.04 (0.909, 1.19)	1.07 (0.915, 1.24)

Supplemental Figures

Supplemental Figure S1. Mean Plasma Concentration-Time Profiles of TBI-223-M2 after Single Doses of TBI-223 Oral Suspension, Capsule, and Tablet Administered under Fasted and Fed Conditions on Linear and Semi-Logarithmic Scales

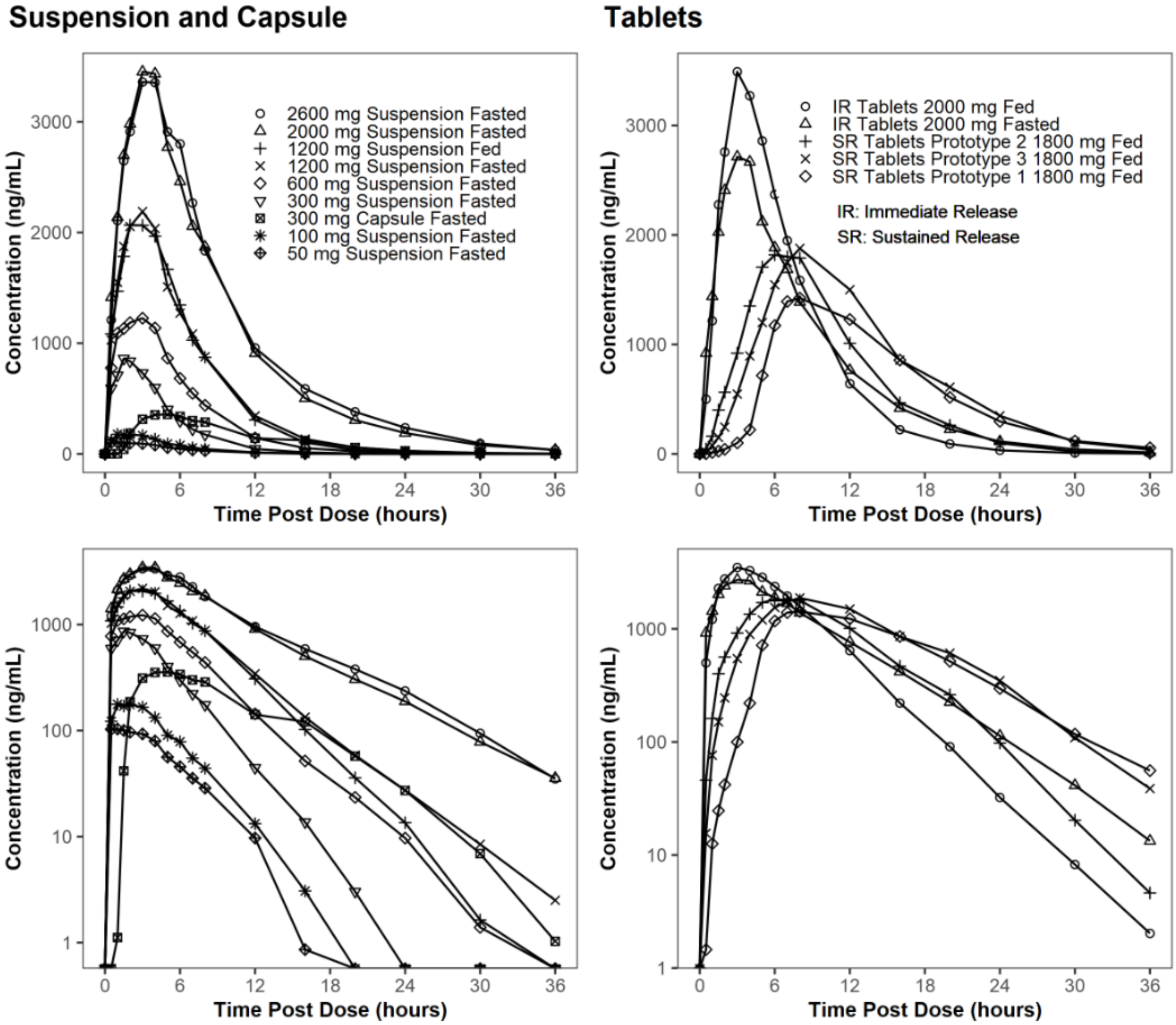

Supplemental Figure S2. Dose Proportionality Plot for C_{max} Following Single Dose Administrations of the Oral Suspension of TBI-223 under Fasted Conditions

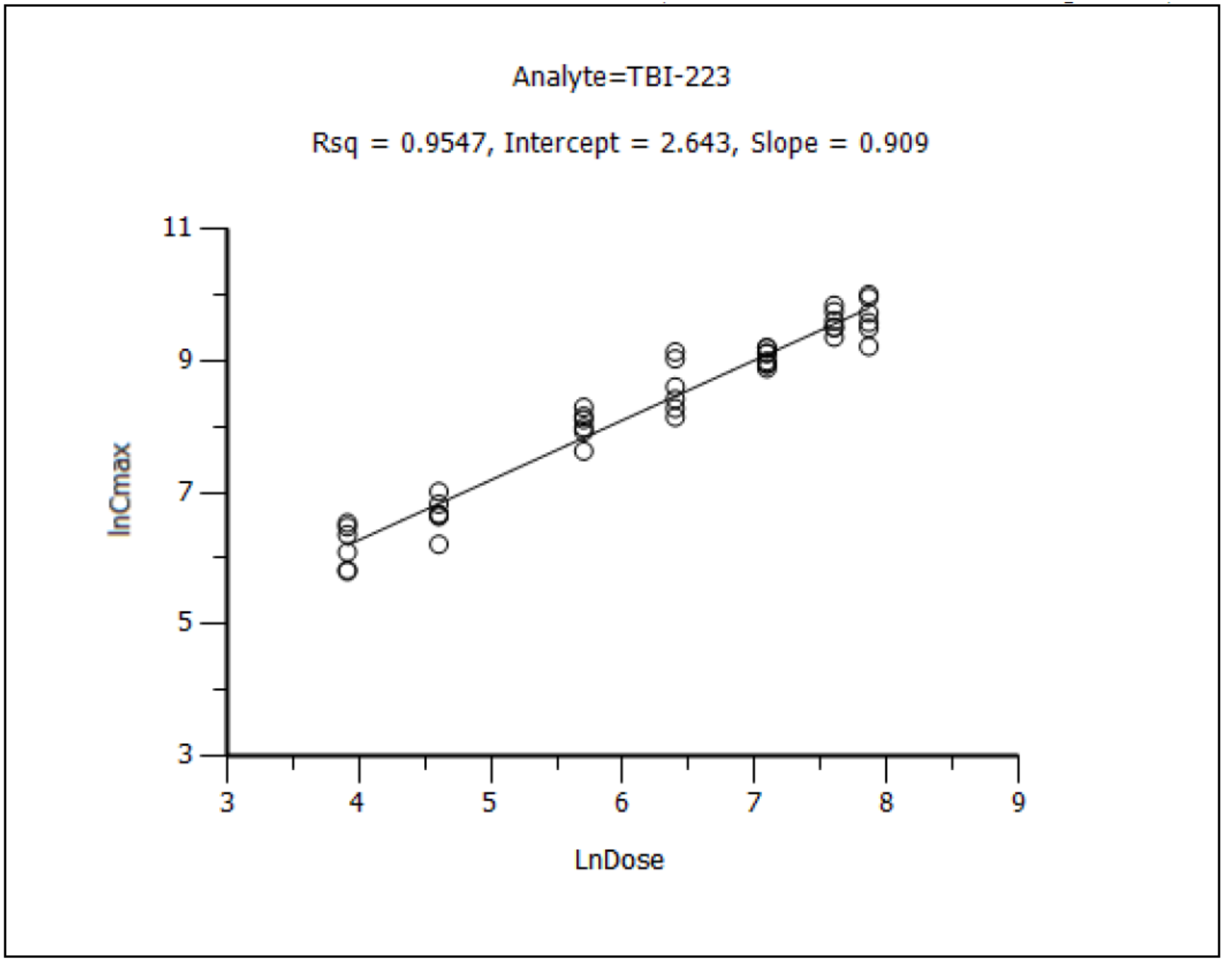

Supplemental Figure S3. Dose Proportionality Plot for $AUC_{0\text{-}t}$ Following Single Dose Administrations of the Oral Suspension of TBI-223 under Fasted Conditions

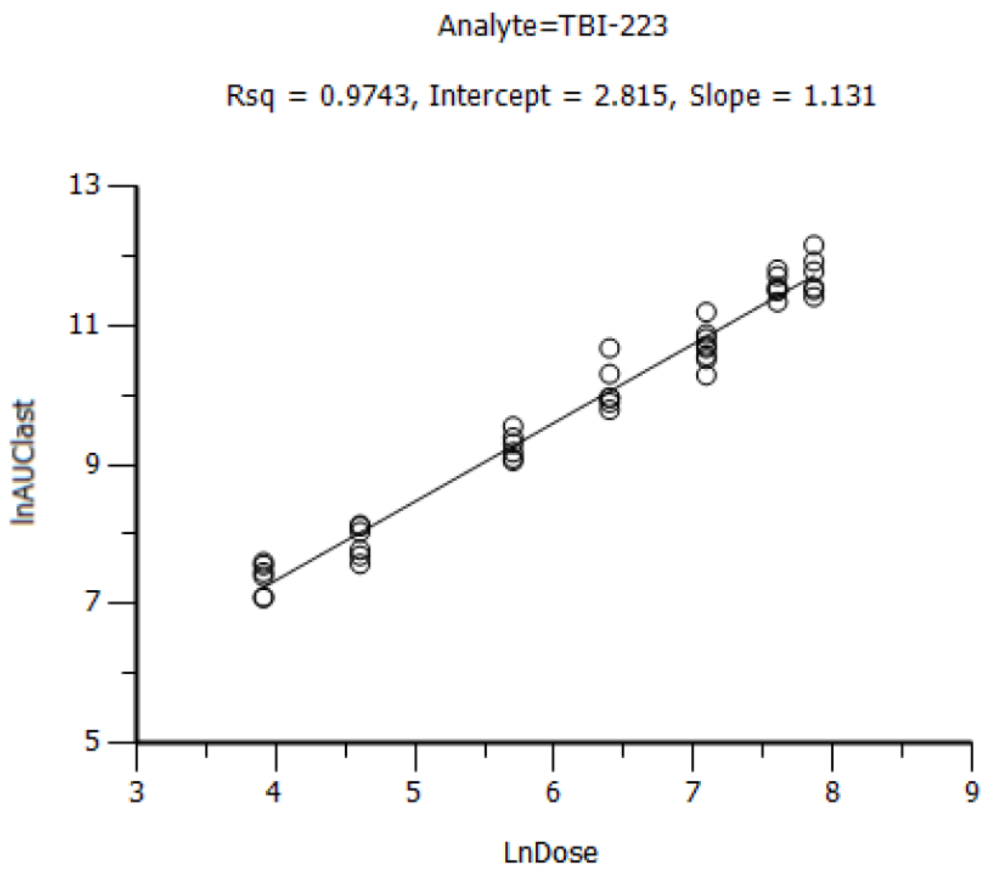

Supplemental Figure S4. Dose Proportionality Plot for $AUC_{0\text{-inf}}$ Following Single Dose Administrations of the Oral Suspension of TBI-223 under Fasted Conditions

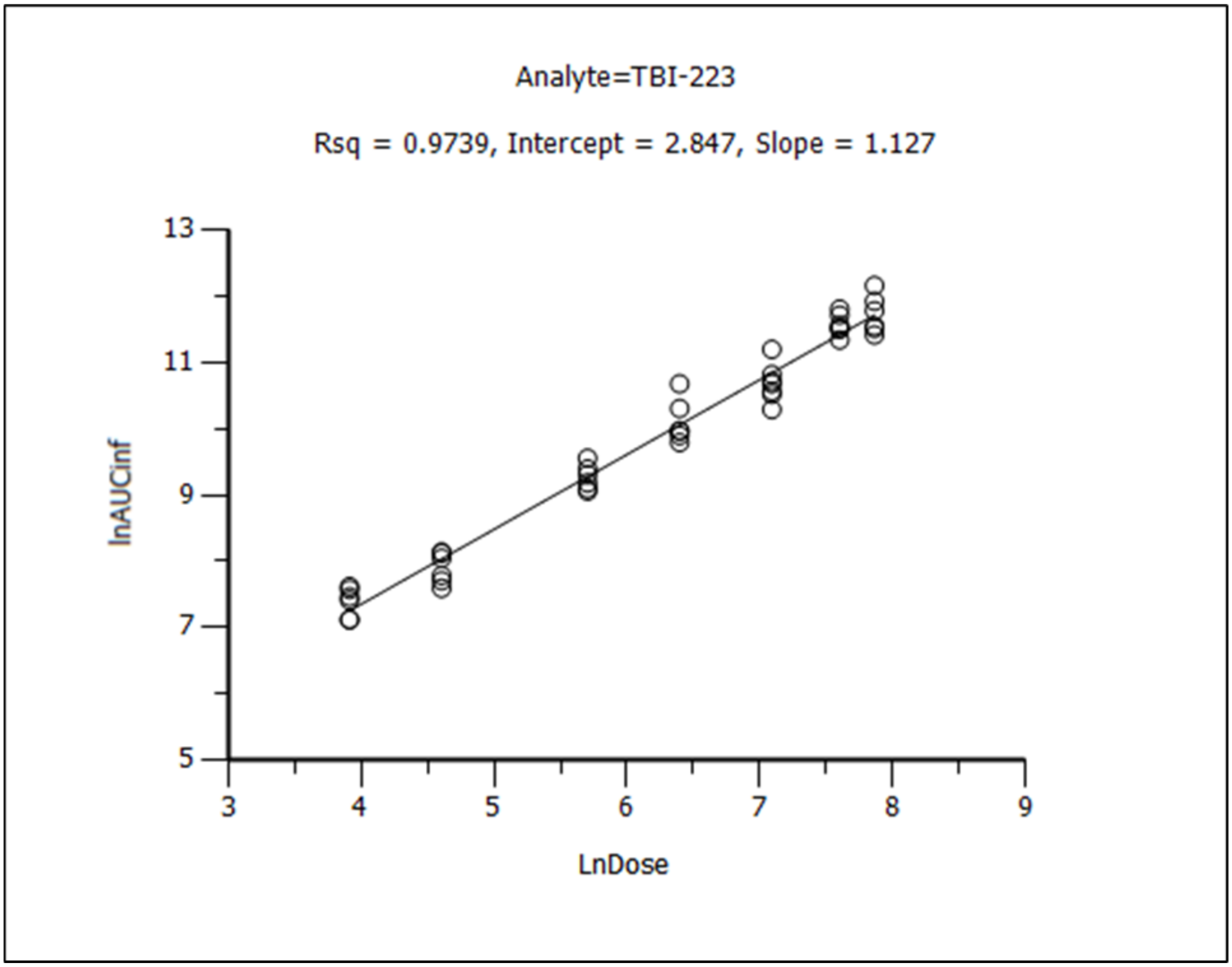

2Supplemental Figure S5. Mean Plasma Concentration-Time Profiles of TBI-223-M2 after Single and Multiple Doses of TBI-223 in MAD Study CL-002 by Treatment, Group, and Day

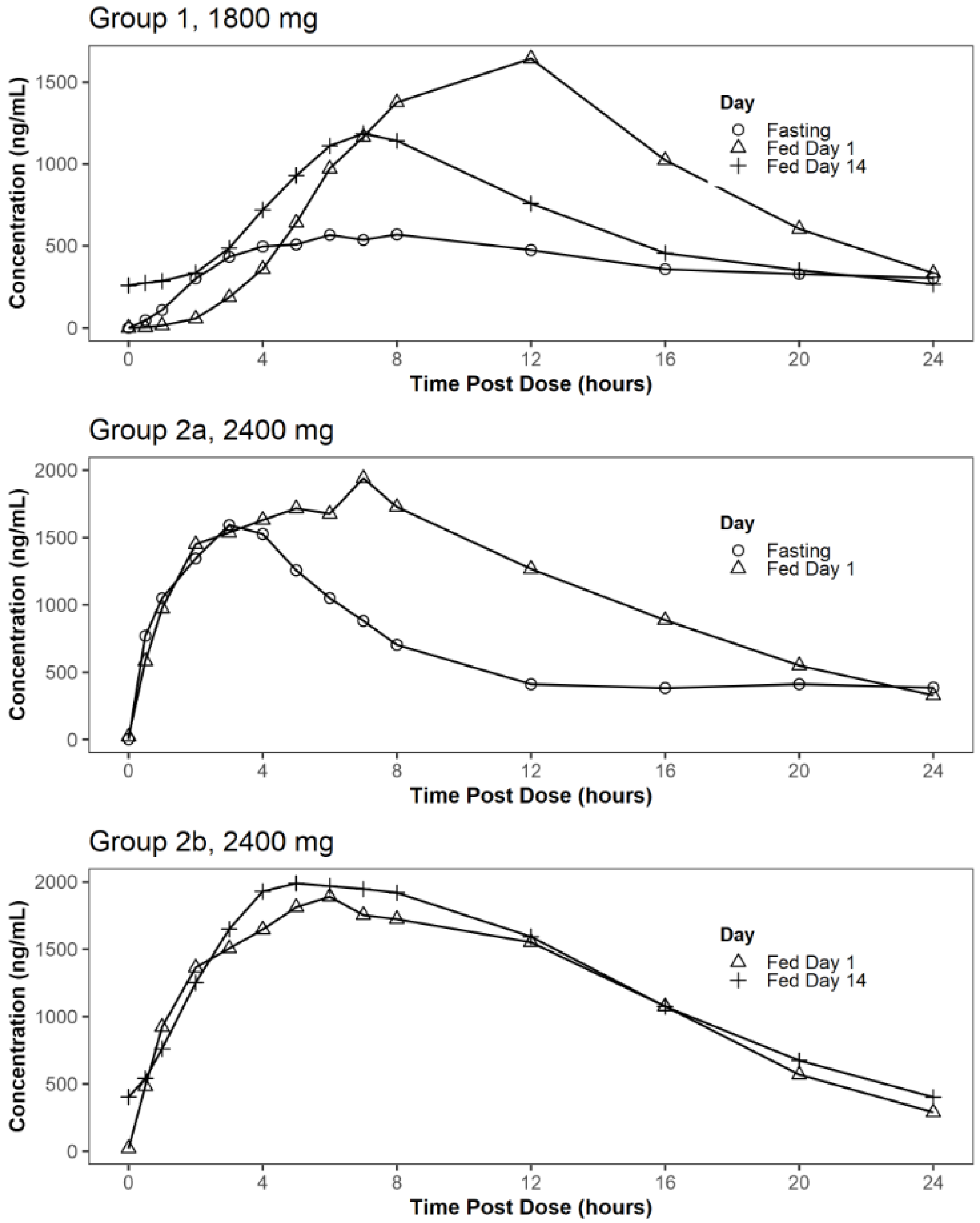

Supplemental Figure S6. Scatter plot of observed plasma concentrations of TBI-223 and estimated placebo-adjusted ΔQTcF, Study CL-001

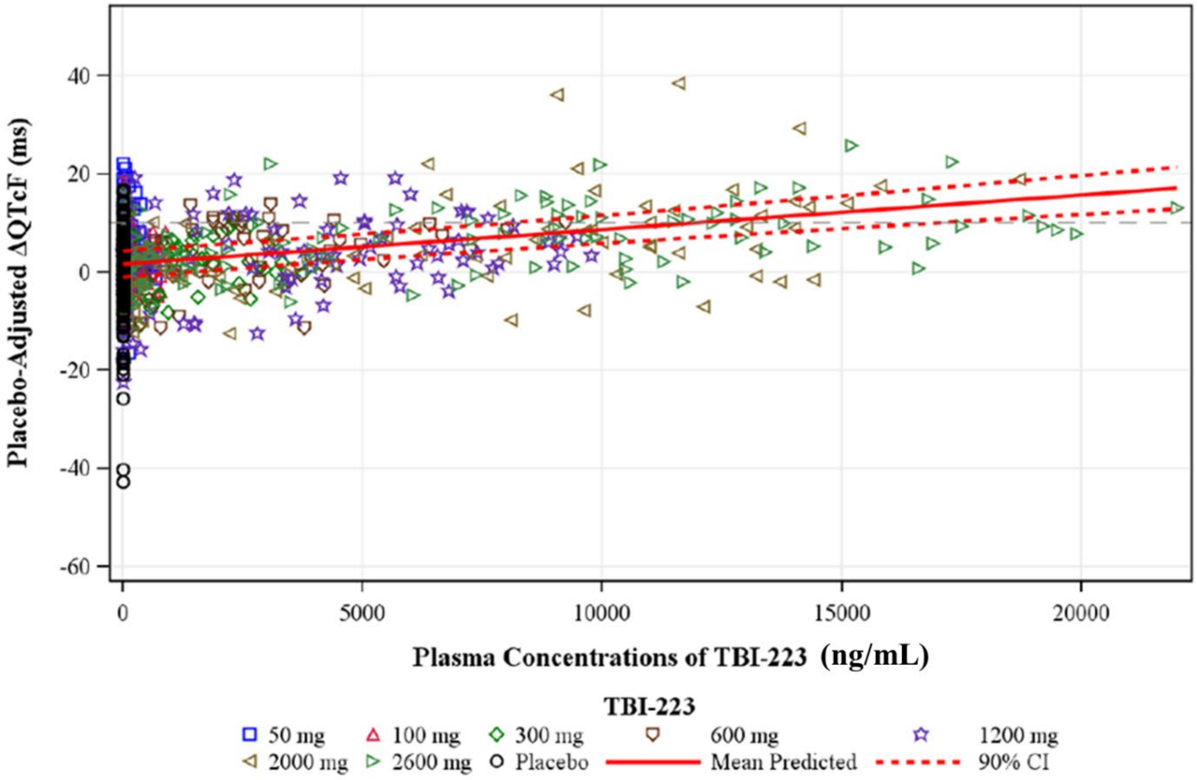

The solid red line with dashed red lines denotes the model-predicted mean ΔΔQTcF with 90% CI, which is calculated from the equation ΔΔQTcF (ms) = 1.56 (ms) + 0.00070 (ms per µg/L) × plasma concentration of TBI-223 (µg/L).

The plotted points denote the pairs of observed drug plasma concentrations and estimated placebo-adjusted ΔQTcF (ΔΔQTcF) by participants for each active dose group and placebo group. The individually estimated placebo-adjusted $\Delta QTcF_{i,k}$ ($\Delta\Delta QTcF_{i,k}$) equals the individual $\Delta QTcF_{i,k}$ for subject $_i$ administered with active drug or placebo at time point $_k$ minus the estimation of the time effect at time point $_k$.
